# Biogenic Silica as a Direct Sol–Gel Precursor for High-Efficiency MSU-X Mesostructure Assembly: Closing the Loop from Rice Husk Waste to Functional Wormhole Frameworks

**DOI:** 10.3390/nano16120748

**Published:** 2026-06-15

**Authors:** Ngo Ha-Son, Le Van-Duong, Cong Ngoc-Thang, Nguyen Thi-Linh

**Affiliations:** 1 Department of Oil Refining and Petrochemistry, Hanoi Unversity of Mining and Geology, 18 Vien Street, Dong Ngac Ward, Hanoi 100000, Vietnam; ngohason@humg.edu.vn (N.H.-S.); congngocthang@humg.edu.vn (C.N.-T.); 2 AMCA Research Group, Hanoi University of Mining and Geology, 18 Vien Street, Dong Ngac Ward, Hanoi 100000, Vietnam; duong.levan@hust.edu.vn; 3 School of Chemistry and Life Sciences, Hanoi University of Science and Technology, 1 Dai Co Viet, Hanoi 100000, Vietnam

**Keywords:** rice husk ash-derived silica, MSU-X mesoporous silica, biogenic silica valorization, wormhole-like pore architecture, ZIF-8/mesoporous composite for adsorption

## Abstract

Direct utilization of biomass-derived silica in neutral surfactant-templated mesoporous synthesis remains underexplored with respect to mesostructure control and functional integration. High-purity silica extracted from acid-treated rice husk ash (~98.4 wt% SiO_2_) was employed as the sole precursor in a fluoride-assisted sol–gel route to synthesize MSU-X frameworks without chemical modification. Systematic parametric variation—pH, Si/surfactant ratio, hydrothermal temperature, and aging duration—establishes quantitative structure–processing correlations. Under optimized conditions (pH 2, Si/Tergitol = 8, 60 °C, 96 h), the resulting material exhibits a wormhole-like mesoarchitecture with a BET surface area of 816 m^2^ g^−1^, mean pore diameter of ~3.6 nm, and three-dimensionally interconnected channels, confirmed by SAXS, TEM, and N_2_ sorption. EDXRF analysis confirms effective impurity removal and high silica incorporation efficiency (~95–96%); thermal stability persists to 700 °C, with incipient crystallization near 800 °C. As a functional demonstration, MSU-X served as an anti-agglomeration scaffold for ZIF-8 crystallization during DDT adsorption. Despite attenuated kinetics relative to pristine ZIF-8—where severe agglomeration occludes active imidazole nodes—the Z8/MSU-X composite achieved near-quantitative DDT removal (74.10 mg g^−1^). This performance stems from the mesoporous matrix driving size-confined, highly dispersed ZIF-8 growth, thereby maximizing active-site exposure. Operating within a reagent-limited regime rather than a capacity-saturated boundary, this efficient depletion confirms that the scaffold successfully suppresses site loss. Ultimately, these findings validate biogenic silica as a directly integrable precursor for tailored mesostructure assembly, positioning agricultural waste as a high-performance feedstock for hierarchical adsorption architectures.

## 1. Introduction

Mesoporous silica materials occupy a central position in advanced materials science by virtue of their tunable pore architectures (2–50 nm), exceptionally high specific surface areas, and chemically versatile frameworks—properties that underpin their widespread deployment in heterogeneous catalysis, drug delivery, molecular adsorption, and environmental remediation [1,2,3]. Among the family of mesoporous silicas, MCM-41, SBA-15, and MSU-X represent the three most extensively studied archetypal frameworks, each distinguished by its templating chemistry and pore topology. MCM-41 forms via an electrostatic S^+^I^−^ mechanism in which cationic alkylammonium surfactants direct the condensation of anionic silicate species into well-ordered two-dimensional hexagonal arrays [1]. SBA-15, synthesized under strongly acidic conditions using amphiphilic triblock copolymers, yields thick-walled, highly ordered hexagonal mesopores with coexistent microporosity [4,5]. MSU-X, by contrast, assembles through a neutral hydrogen-bonding pathway (S^0^I^0^) in which non-ionic polyoxyethylene-type surfactants—including Tergitol, Brij, and Triton families—template silicate oligomers into three-dimensionally connected wormhole-like networks under near-neutral to mildly acidic conditions [6,7,8]. This wormhole architecture, characterized by randomly interconnected channel bifurcations rather than long-range hexagonal periodicity, confers unique advantages: isotropic mass transport, resistance to pore blockage, and enhanced accessibility for large guest molecules—attributes of particular relevance to composite functional materials and environmental applications [7].

The sol–gel method remains the cornerstone synthetic route for mesoporous silica materials, offering exceptional control over structural properties through careful manipulation of hydrolysis and condensation kinetics [9,10]. This versatile approach enables precise tuning of pore architecture, surface area, and framework connectivity by adjusting critical parameters including pH, temperature, precursor concentration, and aging duration [9,11,12,13,14]. In the specific context of MSU-X synthesis, acid-catalyzed condensation at pH ≈ 2, facilitated by fluoride ions (NaF) as a mineralizing agent that accelerates silica network crosslinking, combined with non-ionic Tergitol 15S12 surfactant—a secondary alcohol ethoxylate whose tunable hydrophilic–lipophilic balance and C_12_–C_15_ alkyl chain favor well-defined cylindrical micelle formation—has been shown to produce wormhole frameworks with optimal mesostructural order [6,7]. Recent advances in sol–gel chemistry have refined reaction pathways to achieve enhanced structural uniformity, reduced synthesis times, and improved reproducibility—attributes essential for scalable production of ordered mesoporous frameworks such as MCM-41, SBA-15, and MSU-X [15,16,17,18]. However, there is limited systematic evidence evaluating whether conventional sol–gel synthesis conditions remain sufficient when transitioning from reagent-grade silica to less pure biomass-derived precursors, leaving open the question of whether additional processing steps or modified reaction environments are required.

In parallel, the exploration of renewable biomass-derived silica precursors, notably rice husk ash (RHA), has emerged as a paradigm shift, merging sustainability with advanced material fabrication [19,20]. Rice husk, a globally abundant agricultural residue generated at approximately 150 million tonnes annually, contains up to 90–98 wt% amorphous SiO_2_ following controlled calcination, providing a rich, low-cost source for synthesizing mesoporous silica nanostructures [19,21]. State-of-the-art research has demonstrated that RHA-derived silica can be processed through optimized thermal and chemical treatments to yield high-purity precursors conducive to synthesizing MCM-41, SBA-15, and bifunctional bimodal mesoporous silica with highly ordered pore arrangements and surface areas surpassing 500 m^2^/g [17,22]. These materials have shown competitive or enhanced properties relative to those synthesized from conventional silica sources, including superior catalytic activity, increased hydrogen storage capacity, and efficient adsorption capabilities for environmental pollutants [23,24,25]. Nevertheless, while RHA-derived MCM-41 and SBA-15 have received considerable attention [15,17,22], the synthesis of MSU-X specifically from biogenic silica feedstocks has remained scarcely investigated. The existing literature provides no systematic evaluation of whether the neutral S^0^I^0^ assembly pathway—which is inherently sensitive to silicate speciation and precursor reactivity—can accommodate the compositional heterogeneity intrinsic to biomass-derived precursors without compromising wormhole mesostructure formation.

Notwithstanding these advances, three interconnected knowledge gaps persist and collectively impede the translation of biogenic silica into reliably performing MSU-X frameworks. First, there exists no quantitative, parameter-resolved assessment of how the four primary sol–gel variables—pH, Si/surfactant molar ratio, hydrothermal temperature, and aging duration—govern mesostructure development when RHA-derived sodium silicate, rather than tetraethylorthosilicate (TEOS) or reagent-grade sodium silicate, serves as the precursor. Second, the mass conversion efficiency achievable when employing biogenic silica in MSU-type templated synthesis has not been reported; most studies address structural properties without quantifying the fraction of precursor silica incorporated into the final mesostructured product, leaving the economic viability of such routes unestablished [12,26,27,28]. Third, challenges in ensuring batch-to-batch uniformity and reproducibility of silica quality from heterogeneous biomass sources remain insufficiently resolved, with limited mechanistic guidance on how precursor impurities—particularly residual alkali and alkaline earth metal oxides—affect surfactant–silicate interfacial assembly and, consequently, pore architecture [3,9,27,29,30]. Addressing these gaps is essential for establishing reliable fabrication pathways that can accommodate variability in precursor quality while delivering consistent mesostructural performance aligned with circular-economy objectives [27,28,31,32].

Beyond the synthesis of MSU-X itself, the mesoporous wormhole architecture offers a structurally compelling platform for anchoring functional secondary frameworks. Zeolitic imidazolate framework-8 (ZIF-8)—a sodalite-topology metal–organic framework constructed from Zn^2+^ nodes bridged by 2-methylimidazolate linkers—exhibits pronounced hydrophobicity and high affinity for persistent organic pollutants (POPs) owing to its methylated imidazolate-lined cavity surface [33,34]. However, the intrinsic microporosity of ZIF-8 (pore aperture ~3.4 Å, cavity ~11.6 Å) imposes diffusion limitations for bulky organochlorine molecules such as dichlorodiphenyltrichloroethane (DDT), restricting the utilization of its internal adsorption sites [35]. MSU-X, with its three-dimensionally interconnected mesopore network (3–6 nm), offers a structurally complementary scaffold: by nucleating sub-crystalline ZIF-8 seeds directly onto MSU-X pore walls, the mesoporous transport network can be expected to overcome the intraparticle diffusion bottleneck of the parent microporous framework, thereby enhancing both the rate and total capacity of DDT uptake. The design and validation of such Z8/MSU-X composites thus provide an application-driven context in which the structural quality of the RHA-derived MSU-X support—particularly pore connectivity, surface area, and thermal stability—directly governs functional performance.

The present work addresses these interconnected gaps through a systematic, evidence-based approach. Specifically, we investigate the effects of hydrothermal aging time, temperature, pH, and Si/Tergitol 15S12 molar ratio on MSU-X formation from acid-leached rice husk ash via fluoride-assisted sol–gel synthesis, and establish quantitative relationships between synthesis conditions and the resulting wormhole mesostructure. EDXRF compositional tracking across the full synthesis sequence—from raw RHA through calcined MSU-X—provides, for the first time in this material system, a rigorous mass conversion efficiency assessment, demonstrating that ~95.6% of available biogenic SiO_2_ is incorporated into the final mesostructured product. The structural and textural benchmarks of the RHA-derived MSU-X are evaluated against state-of-the-art MSU-X produced from reagent-grade precursors, confirming structural parity without modification of synthesis conditions. Finally, the utility of MSU-X as a high-performance support is demonstrated through the preparation and evaluation of Z8/MSU-X composites for DDT adsorption, providing mechanistic insight into the complementary roles of mesoporous transport and microporous adsorption in composite functional materials. Together, these contributions establish a scientifically rigorous and scalable pathway for converting agricultural silica waste into high-value mesoporous frameworks within a circular-economy manufacturing context [36,37,38].

## 2. Materials and Methods

### 2.1. Materials

The rice husk utilized in this study was procured as an agricultural byproduct from a commercial rice milling facility located in Thái Bình Province, Vietnam. Analytical-grade chemicals, including sodium hydroxide (NaOH, 98%), hydrochloric acid (HCl, 37%), and sodium fluoride (NaF, 98%), were sourced from Deajung, Gyeonggi-do, Republic of Korea. High-purity reagents such as Tergitol 15S12 (≥99%), zinc nitrate hexahydrate (Zn(NO_3_)_2_·6H_2_O, 98%), ammonia solution (NH_4_OH, 25%) and methanol (MeOH, CH_3_OH, 99.9%) were purchased from Sigma-Aldrich, Ho Chi Minh City, Vietnam; 2-methylimidazole (2-Mim, C_4_H_6_N_2_, 99%) was purchased from Thermo scientific, Waltham, MA, USA; and dichlorodiphenyltrichloroethane (DDT, C_14_H_9_Cl_5_, 99.5%) was purchased from Chemservice Inc, Worms, Germany. All reactants were utilized without extra purification. All materials were handled under standard laboratory conditions to maintain their integrity and ensure reproducibility of experimental procedures.

### 2.2. Extraction of Silica from Rice Husk

The rice husk was carefully washed several times with deionized water to remove dust and surface impurities. It was then subjected to acid treatment by soaking in a 1 M hydrochloric acid solution at a solid-to-liquid weight ratio of 1:2 under ambient conditions for 12 h to eliminate metallic contaminants. After acid leaching, the material was repeatedly rinsed with deionized water until the wash water reached neutral pH. The neutralized rice husk was dried in an oven at 105 °C for two hours to remove moisture. Subsequently, it was calcined in air at 600 °C for three hours to decompose organic matter, resulting in white rice husk ash rich in amorphous silica. The ash was then reacted with a 5 M sodium hydroxide solution with a solid-to-liquid ratio of 1:8 (*w*/*v*) under reflux at 150 °C for three hours to solubilize silica as sodium silicate, which was used as a precursor for subsequent synthesis steps. This comprehensive preparation ensures the removal of impurities and maximizes silica extraction for high-quality mesoporous silica production.

### 2.3. Synthesis of MSU-X

A solution of 1.2 g Tergitol 15S12 was prepared by dissolving it in 100 mL of distilled water at ambient temperature. To this surfactant solution, 12.2 mL of 1 M sodium silicate derived from rice husk ash was gradually introduced under gentle stirring maintained at low speed for three hours. The pH of the mixture was then adjusted to approximately 2 by the careful addition of 0.25 M hydrochloric acid, after which the mixture was allowed to stir continuously at room temperature for twelve hours. Subsequently, a 0.5 M sodium fluoride solution was incorporated, and the resulting mixture was subjected to hydrothermal treatment at 60 °C for a duration of 96 h. Upon completion, the solid product was separated by filtration, followed by thorough washing until the filtrate attained neutral pH. The collected solid was dried and calcined at 550 °C for three hours, resulting in a fine, porous white powder—designated as MSU-X—characterized by its well-defined mesoporous architecture and high surface area. This protocol, involving controlled pH regulation and fluoride-assisted catalysis during synthesis, effectively governs the nucleation and growth of silica frameworks, yielding materials with precise pore structure and morphology. The detailed formulation and parametric variations employed for the synthesis of the differentiated MSU-X frameworks are consolidated in Table 1.

### 2.4. Synthesis of ZIF-8

ZIF-8 was synthesized via the following procedure. Zn(NO_3_)_2_·6H_2_O (1 mmol) was dissolved in methanol (10 mL) to yield Solution A, while 2-Mim (4 mmol) was dissolved separately in methanol (10 mL) to yield Solution B. Solution A was added dropwise to Solution B under continuous magnetic stirring until complete dissolution was achieved and the mixture adopted a milky-white appearance. The resulting suspension was then allowed to crystallize statically at 60 °C for a minimum of 6 h. Upon completion of the reaction, the ZIF-8 powder was recovered by centrifugation at 5000 rpm for 20 min, followed by three successive washing cycles with methanol to remove residual reactants. The purified product was subsequently dried under vacuum at a reduced pressure of 4 mmHg and a temperature of 70 °C until a constant mass was obtained.

### 2.5. Preparation of the Z8/MSU-X Composite

In this study, a ZIF-8-functionalized MSU-X mesoporous silica composite was prepared via a post-synthetic modification strategy, whereby ZIF-8 crystallites were grown in a controlled manner directly onto the inner pore walls of the MSU-X support matrix.

Briefly, 2-Mim was dissolved in methanol in a reaction vessel pre-loaded with 0.3 g of MSU-X. In a separate vial, Zn(NO_3_)_2_·6H_2_O was dissolved in methanol and the resulting solution was introduced dropwise into the MSU-X-containing vessel under magnetic stirring for 10 min to ensure homogeneous dispersion. Solution A was subsequently added to the mixture, and stirring was continued for an additional 10 min. Crystallization of ZIF-8 onto the MSU-X surface was carried out at 60 °C for 4 h under gentle agitation. Where applicable, auxiliary mineralizing agents—aqueous ammonia (NH_4_OH)—were introduced during the crystallization step to modulate crystal nucleation and growth. Following the reaction, the composite product was isolated by vacuum filtration and washed three times with methanol to remove unreacted precursors and by-products. The washed solid was dried under vacuum at 4 mmHg and 70 °C for 6 h. The resulting material is hereafter designated Z8/MSU-X.

### 2.6. Material Characterizations

The elemental purity of the Rice Husk (RH) and Rice Husk Ash (RHA) was quantitatively determined by Energy Dispersive X-ray Fluorescence (EDXRF) spectroscopy (Oxford Instruments, Scotts Valley, CA, USA). Measurements were conducted using a Silicon drift detector (Si drift) under vacuum conditions, which is essential for accurately quantifying light and trace elements. The X-ray tube utilized a Rhodium target (as inferred from the spectral data) and was operated at 30.0 kV and 100.0 A for a real time of 113.9 s, enabling precise quantitative assessment of both major and minor constituents. In addition, Fourier-transform infrared spectroscopy (FTIR) spectra were recorded to provide information on functional groups and bonding environments using an Affinity-1S spectrometer (Shimadzu, Kyoto, Japan). The samples were ground with KBr powder and pressed into 1 mm pellets for FTIR measurement in the frequency range 400–4000 cm^−1^. Mesostructural ordering of the synthesized MSU-X was assessed by small-angle X-ray scattering (SAXS) employing Cu Kα radiation (λ = 1.5406 Å) under 40 kV and 40 mA on a Bruker D8 Advance diffractometer (Bruker, Berlin, Germany). Patterns were collected over a 2θ range of 0.5° to 10° at a scan rate of 0.01° s^−1^ to characterize periodicity within the mesoporous framework. Transmission electron microscopy (TEM) images were captured using a JEOL 3010 microscope (Jeol, Tokyo, Japan) operating at a voltage between 80 and 300 kV. The scanning electron microscopy (SEM) images were obtained using a S-4800 microscope (Hitachi, Tokyo, Janpan), with lattice resolution as fine as 0.14 nm and magnifications from 50,000× to 200,000× to reveal pore morphology and particle structure. Nitrogen adsorption–desorption isotherms were measured at 77 K with a Micromeritics ASAP 2060 (Micromeritics, Norcross, GA, USA) after degassing samples at 150 °C for 12 h. Surface area calculations employed the Brunauer–Emmett–Teller (BET) method. Pore size distribution was derived by the Barrett–Joyner–Halenda (BJH) method from the adsorption branch, and total pore volume was determined at a relative pressure of 0.99. Thermal stability and phase transitions were examined via thermogravimetric analysis (TGA) under nitrogen flow, with temperature ramped at 10 °C/min up to 1000 °C using a SETARAM analyzer (KEP technologies, Austin, TX, USA). This combination of complementary techniques provided a thorough characterization confirming the structural integrity, porosity, composition, and thermal robustness of MSU-X.

### 2.7. Adsorption Performance Evaluation

The adsorption performance of Z8/MSU-X toward DDT was assessed as follows: a stock solution of DDT (25 mg L^−1^) was prepared in a binary water/acetone solvent system (*v*/*v* = 9:1). Aliquots of 15 mL of this solution were transferred into individual test tubes, each containing 5 mg of Z8/MSU-X. The suspensions were agitated on a Biobase orbital shaker at 700 rpm for predetermined time intervals to evaluate adsorption kinetics.

At each designated time point, shaking was ceased and the suspension was centrifuged at 4000 rpm for 5 min to separate the adsorbent from the solution phase. A 2 mL aliquot of the supernatant was withdrawn and passed through a syringe filter to remove residual particulates. The filtrate was subsequently subjected to liquid–liquid extraction using n-hexane. Following a settling period of 5 min to allow complete phase separation, the upper organic layer containing the DDT/n-hexane fraction was carefully collected, dried, and submitted for quantitative analysis by GC-MS.

The time-dependent adsorption capacity of Z8/MSU-X, q_t_ (mg g^−1^), was calculated according to the following equation:

$$q_{t}\;=\;\frac{(C_{0}\;-{\;C}_{t})\cdot V}{m}$$

where C_0_ and C_t_ (mg L^−1^) denote the initial DDT concentration and the residual DDT concentration at time t, respectively; V (L) is the volume of the DDT solution; and m (g) is the mass of Z8/MSU-X employed.

The concentrations of DDT in solution prior to and following adsorption were determined using an Agilent 5977B GC-MS system (Agilent Technologies, Santa Clara, CA, USA) operated in selected ion monitoring (SIM) mode. Chromatographic separation was performed on a DB-5 capillary column (30 m × 0.32 mm × 0.25 μm) using ultrahigh-purity helium as the carrier gas at a constant flow rate of 1.5 mL min^−1^. The oven temperature program was as follows: isothermal hold at 40 °C for 10 min, ramp to 200 °C at 20 °C min^−1^, followed by a final ramp to 280 °C at 10 °C min^−1^.

## 3. Results and Discussion

### 3.1. Raw Materials Treatment Efficiency

The physicochemical characterization of rice husk (RH) and its calcined derivative (RHA) was carried out using FTIR, XRD, and EDXRF analyses (Figures S1 and S2).

FTIR spectra of RH display absorption bands characteristic of cellulose, hemicellulose, and lignin—specifically –OH stretching (~3313 cm^−1^), C–H vibrations (~2949 cm^−1^), C=O stretching (~1634 cm^−1^), and a cellulose-associated Si–O band at 1036 cm^−1^ [39]. Upon calcination at 600 °C, these organic signatures are completely extinguished, and RHA exhibits dominant Si–O–Si asymmetric and symmetric stretching at 1057 and 796 cm^−1^, confirming formation of a condensed siloxane network [40,41]; the concurrent disappearance of –OH and C–H bands confirms substantial removal of organic constituents, consistent with prior reports on biomass-derived silica [42,43].

XRD corroborates this transformation: RH presents a semicrystalline pattern with a cellulose reflection at 2θ ≈ 16.2° (PDF 03-0289) superimposed on a broad amorphous SiO_2_ halo at 2θ ≈ 22.4° (PDF 29-0085, JCPDS/ICDD) and a weak quartz feature near 2θ ≈ 36°, while RHA retains only the broad amorphous silica halo with no residual crystalline organic reflections, confirming complete burnout of the organic matrix.

EDXRF quantification of RHA yields a SiO_2_ purity of 98.44 wt.%, with all metallic oxide impurities (CaO 0.706, SO_3_ 0.685, Fe_2_O_3_ 0.033 wt.%) collectively below 1.6 wt.%. The low concentrations of residual alkali, alkaline earth, and transition metal oxides confirm the efficacy of the HCl acid-leaching and thermal calcination protocol, in agreement with established purification benchmarks for agricultural biomass silica [44,45].

This purity level is further consistent with systematic assessments of acid-treated, calcined rice husk ash as a competitive precursor for high-performance catalyst supports and adsorbents [21,46] and meets the threshold required for advanced mesoporous silica synthesis where metal impurity tolerance is minimal.

### 3.2. Optimization of MSU-X Synthesis Conditions

Mesoporous materials exhibit ordered arrangements of voids on the nanoscale, distinguishing their framework by spatial periodicity; however, the silica walls themselves do not display long-range atomic order and are inherently amorphous. The regularity observed in the pore distribution arises from templated synthetic routes, resulting in a well-defined arrangement that can be interrogated using small-angle X-ray scattering (SAXS) or low-angle X-ray diffraction (XRD) methods. These techniques, which probe the scattering intensity within 2θ angles below 10°, allow for the identification of periodic mesostructure without indicating crystallinity in the pore walls [18,47].

From a crystallographic perspective, this dichotomy stems from the ability of self-assembled surfactant templates to induce uniform spacing and symmetry across mesoporous networks, providing crystal-like order at the mesoscale—yet the absence of a regular atomic lattice within the wall material precludes classification as crystalline. Detailed SAXS analysis captures this mesoscale ordering, while the lack of sharp Bragg reflections in wide-angle XRD corroborates the amorphous nature of the framework [48]. Nitrogen physisorption and electron microscopy serve as complementary methods to resolve pore size distributions and visualize the architecture, validating the presence of uniform channels and identifying defects or compositional heterogeneity [18].

Optimization of synthesis conditions for MSU-X materials often relies on systematic experiments varying composition, pH, aging times, and thermal protocols, with SAXS providing rapid feedback on mesostructural development. Structural outcomes are routinely compared across parallel batches to elucidate the influence of individual synthesis parameters, guiding iterative adjustments aimed at maximizing order while minimizing wall defects [49,50].

Altogether, the ordered pore architecture and lack of atomic periodicity in mesoporous materials necessitate precise characterization using suitable techniques; findings at the nanoscale inform process refinements and establish the correlation between synthetic conditions and functional material properties.

#### 3.2.1. Effect of Aging Time

**Figure 1:**
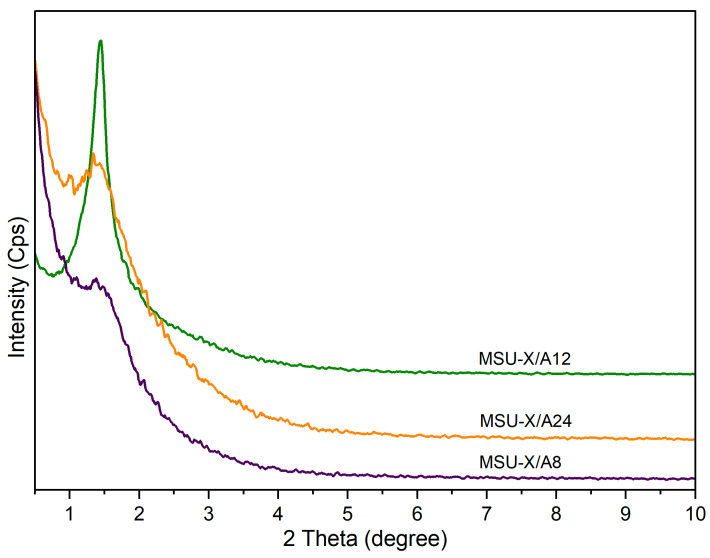
SAXS patterns of MSU-X material samples at different aging times.

The aging time plays a pivotal role in governing the polymerization of TO_4_ tetrahedra within the MSU-X mesoporous silica framework, particularly when rice husk ash serves as the silica precursor. Narrow-angle X-ray diffraction (XRD) analysis shown in Figure 1 reveals that, at an aging duration of 12 h, a distinct, sharp peak emerges at 2θ ≈ 1.4°, corresponding to the (100) reflection of disordered mesoporous materials [6]. This observation indicates the formation of a regularly spaced pore network characteristic of uniform mesostructures, indicative of sufficient SiO_4_^4−^ tetrahedral polymerization at this timescale [51,52,53].

At the molecular level, sodium silicate undergoes hydrolysis and rearrangement in aqueous or alkaline environments, yielding monomeric Si(OH)_4_ or tetrahedral silicate anions (SiO_4_^4−^):

Na_2_SiO_3_ → 2Na^+^ + SiO_3_^2−^

SiO_3_^2−^ + OH^−^ + H_2_O → SiO_4_^4−^ + H_3_O^+^

Polymerization proceeds via condensation of these monomers into silicate oligomers, forming dimers (Si_2_O_7_^6−^), trimers (Si_3_O_10_^8−^), and hexamers (Si_6_O_18_^12−^) through shared oxygen bridges, constructing the basis of an interconnected tetrahedral network critical for mesostructure formation. The simplified condensation reaction between monomers exemplifies this process [51,54]:

2 Si(OH)_4_ → (HO)_3_Si–O–Si(OH)_3_ + H_2_O

(HO)_3_Si–O–Si(OH)_3_ + Si(OH)_4_ → [(HO)_3_Si–O–Si(OH)_2_–O–Si(OH)_3_] + H_2_O

Insufficient aging (e.g., 8 h) curtails the extent of tetrahedral condensation, preventing significant oligomer formation and resulting in the absence of distinct XRD mesophase peaks. Extended aging beyond 12 h (e.g., 24 h) leads to the appearance of broader, less intense peaks. Prolonged hydrothermal aging results in the formation of excessively polymerized silicate oligomers, which present kinetic and steric barriers to efficient condensation at the surfactant-silica interface. These observations align with theoretical and spectroscopic insights into silica polymerization pathways, underscoring the integral role of controlled aging in achieving optimal mesoporous architecture [52,55].

This interplay between monomer hydrolysis, oligomerization, and polymer network assembly elucidates the temporal window in which ordered mesostructure formation is maximized, with aging serving as a tunable parameter to balance nucleation and growth kinetics, ultimately influencing pore uniformity and wall integrity critical for targeted applications [54].

#### 3.2.2. Effect of Hydrothermal Temperature

**Figure 2:**
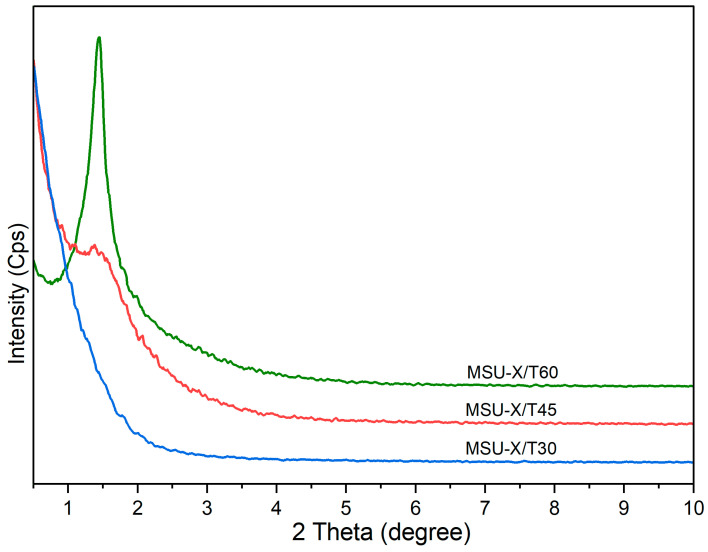
SAXS patterns of MSU-X materials synthesized at different hydrothermal temperatures.

Hydrothermal treatment induces the interaction between silicate oligomers and tubular surfactant micelles, guiding the assembly of mesoporous frameworks through a liquid crystal templating mechanism (Figure 2). In this system, Tergitol, a non-ionic surfactant (S_0_), forms hydrogen bonds with neutral silicate oligomers [(HO)_3_Si–O–Si(OH)_2_–O–Si(OH)_3_] (I_0_), involving hydroxyl groups of silanols and the ethoxy or hydroxyl functionalities of Tergitol. This association results in the encapsulation of micellar templates by silicate species, forming a cohesive silanol (Si–O–Si) shell.

At 60 °C, small-angle X-ray scattering of MSU-X reveals sharp and intense diffraction peaks, signaling well-defined condensation and crystallization of silica induced by surfactant micelles. Conversely, synthesis at 45 °C yields broader, less intense peaks near 2θ = 1.6°, indicating partial network formation and smaller domain sizes due to incomplete polymerization. Below 30 °C, insufficient kinetic energy precludes condensation, preventing mesophase formation.

This temperature-dependent structural evolution highlights the delicate balance between kinetic and thermodynamic controls governing mesoporous silica synthesis, emphasizing the critical role of hydrothermal parameters when employing rice husk ash-derived silica precursors.

#### 3.2.3. Effect of Hydrothermal Time

**Figure 3:**
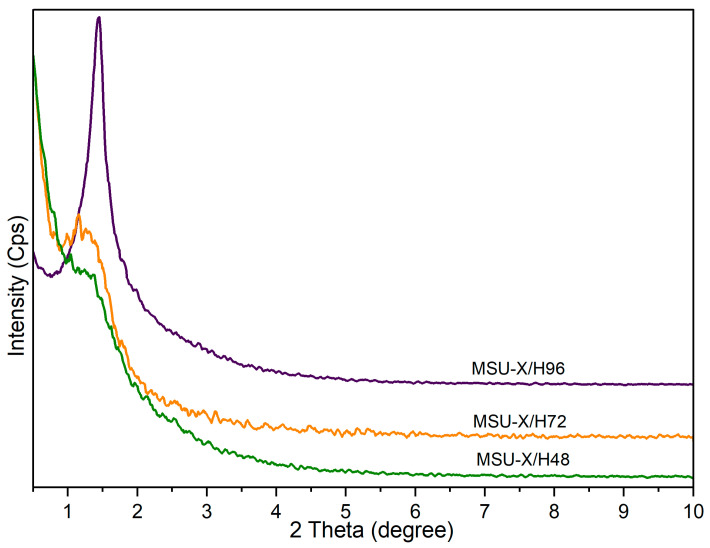
SAXS patterns of MSU-X materials synthesized at different hydrothermal durations.

The effect of hydrothermal time on the MSU-X material samples was evaluated using small-angle X-ray diffraction (XRD) analysis, as shown in Figure 3.

The SAXS profiles elucidate the decisive influence of hydrothermal duration on the mesoporous architecture derived from rice husk silica. At 48 h, the absence of a distinct scattering peak reflects insufficient condensation of silica around surfactant micelles, thus disfavoring formation of an ordered pore network. With 72 h of treatment, a broad, low-intensity peak emerges near 2θ = 1.5°, characteristic of early-stage mesopore development but with incomplete silica network organization—paralleling previous reports on nucleation and evolution of mesoporous domains under suboptimal hydrothermal conditions. Extension to 96 h results in a pronounced, sharp peak at the same angle, demonstrating robust self-assembly and consolidation of the mesoporous framework due to complete silica condensation onto the templating micelles. The appearance of a solitary (100) reflection and lack of higher-order peaks suggests predominantly hexagonal order with local disorder or heterogeneity in pore uniformity, which is frequently linked to the dynamic interplay between surfactant templating and silica precursor evolution [15,56,57].

#### 3.2.4. Influence of Reaction Medium Acidity

**Figure 4:**
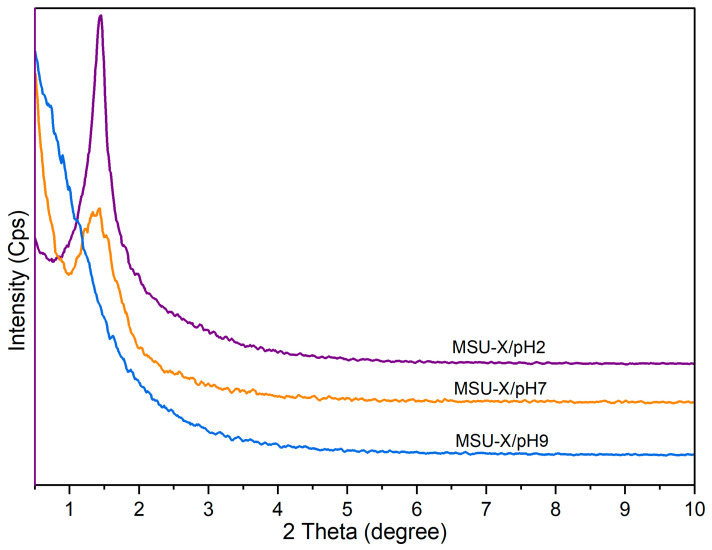
SAXS patterns of MSU-X materials synthesized under different pH conditions.

Figure 4 illustrates the influence of pH on silica crystallization during mesoporous structure formation from rice husk precursors. The pH environment critically modulates gelation and the condensation dynamics of inorganic species around surfactant micelles [58]. While the optimal pH is contingent on the surfactant-inorganic precursor pair, the rice husk-derived silica precursor exhibits gelation and crystallization efficacy across a broad pH range, necessitating precise evaluation under experimental conditions [56,59].

The SAXS patterns reveal mesoporous formation at both acidic (pH = 2) and neutral (pH = 7) conditions. At pH = 2, catalysis by NaF enhances silica condensation and crystallization onto micellar templates, producing sharper and more intense diffraction peaks indicative of a well-ordered framework. Conversely, neutral pH demonstrates mesopore presence but with broader, less intense peaks, reflecting less efficient condensation and partial structural ordering. This contrast aligns with literature that highlights acid catalysis as a key driver for silica network connectivity and mesostructural integrity during synthesis [56,60]. Consequently, subsequent material preparation in this study prioritizes acidic conditions (pH = 2) to ensure robust, stable mesoporous frameworks.

#### 3.2.5. Effect of Si/Ter Molar Ratio

The formation of mesopores from silicon-containing precursors depends on the interaction between neutral silicate oligomers and the number of adsorption centers on Tergitol micelles. The appropriate molar ratio of Si/Ter determines the ability to form mesoporous structures in the material under study.

**Figure 5:**
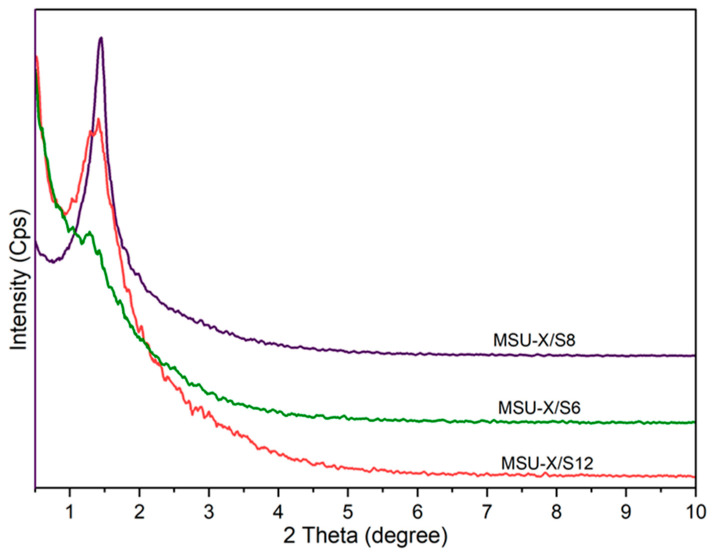
SAXS patterns of MSU-X materials synthesized with different Si/Ter ratios.

Figure 5 shows the small-angle X-ray scattering (SAXS) patterns of MSU-X samples synthesized at a Si/Ter molar ratio of 6. At this ratio, a weak peak appears in the low-angle region (2θ), which, although not very sharp, indicates the initial formation of mesoporous structures from the rice husk-derived silica precursor. This also suggests the beginning of the transformation of Na_2_SiO_3_ from rice husk ash into silicate tetrahedra during gel formation.

When the Si/Ter ratio reaches 8, the development of mesoporous structure is more evident, reflecting a balance between the number of adsorption centers and silicate oligomers. The SAXS results show the formation of mesoporous material, indicated by a sharp peak at 2θ = 1.4°. However, when the Si/Ter ratio is too high, this balance is disrupted, leading to competitive condensation on the surface of Tergitol micelles, resulting in poorly formed mesopores.

The information from the SAXS patterns confirms the feasibility of using rice husk silica as a precursor material for synthesizing mesoporous materials. The presence of a single peak at a low angle indicates that the synthesized MSU-X material has a mesoporous structure with disordered mesopore arrangements. This structure will be further characterized by transmission electron microscopy (TEM), which will be presented in the following section.

### 3.3. Characterizations of MSU-X

#### 3.3.1. Surface Morphology and Material Structure

**Figure 6:**
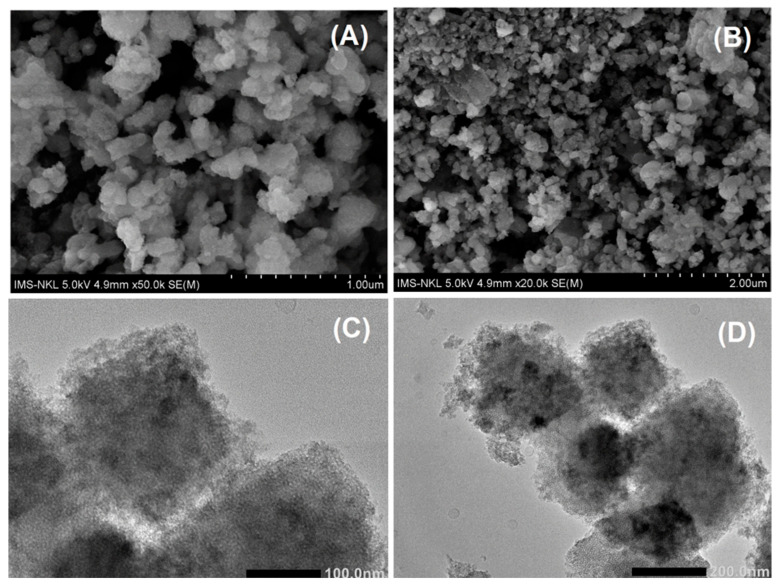
SEM images (**A**,**B**) and TEM images (**C**,**D**) of MSU-X material.

The SEM micrographs (Figure 6A,B) reveal that the MSU-X sample is composed of irregularly aggregated nanoparticles forming secondary clusters with a rough and corrugated surface. The particles exhibit a sponge-like morphology with interconnected granular domains, indicative of a mesostructured framework formed through the condensation of silica around the surfactant micelles. The relatively uniform particle dimensions, in the submicrometer range (~95 ± 15 nm), suggest homogeneous nucleation and growth during synthesis, while the lack of long-range ordering implies the formation of a wormhole-like mesophase typical of MSU-type materials. Such morphology is often associated with open textural porosity and efficient mass transport properties [7].

TEM analysis (Figure 6C,D) further confirms the presence of a disordered mesoporous network. The images clearly show wormhole-like channels intertwined within spherical aggregates, with no evidence of long-range hexagonal ordering as seen in SBA-type materials. The observed mesopores, approximately 3–4 nm in diameter, are uniformly distributed and interconnected, forming a pseudo-three-dimensional pore network. The relatively dense pore walls indicate a high degree of silica crosslinking, which can enhance the mechanical and thermal stability of the framework. The combination of uniform mesopore size, thick walls, and disordered connectivity is characteristic of MSU-X materials, providing an optimal balance between surface accessibility and structural robustness. TEM observations, together with the corresponding XRD features, indicate the presence of a wormhole-like mesostructure in the material. These morphological and structural observations strongly support the successful formation of the intended MSU-X mesophase under the selected synthesis conditions.

#### 3.3.2. Structural Characteristics and Pore Size

**Figure 7:**
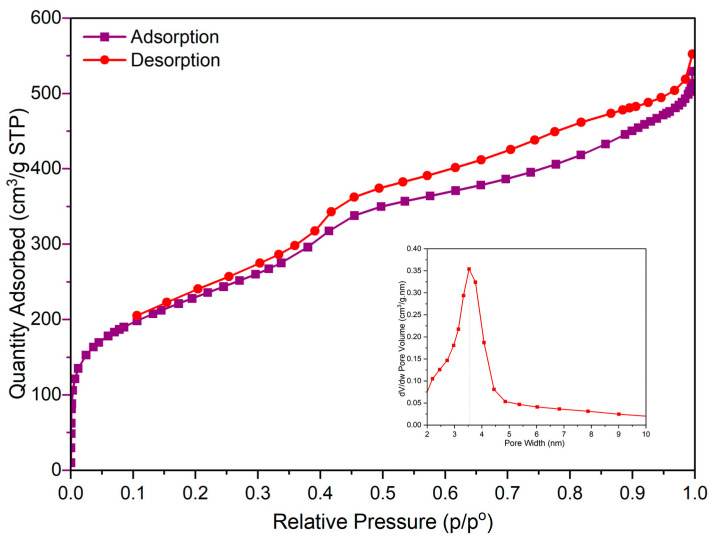
Nitrogen adsorption–desorption isotherm of MSU-X material and pore size distribution curve (inset).

To gain more information about the structure and pore diameter, the study employed the nitrogen adsorption–desorption isotherm method. The obtained results are presented in Figure 7.

A general examination reveals that the nitrogen adsorption–desorption isotherm of the MSU-X sample exhibits the typical shape of a Type IV isotherm, which is characteristic of mesoporous materials according to IUPAC classification. Both the adsorption and desorption branches of the N_2_ isotherm display an initial increase in adsorption capacity at low relative pressure (P/P_0_ < 0.1). This phenomenon is attributed to the transition from monolayer adsorption on the pore surface to multilayer adsorption on the surface of the mesoporous material. This indicates that, in addition to monolayer adsorption on the mesoporous surfaces, there may also be monolayer adsorption occurring in micropores, leading to an increase in monolayer adsorption capacity. Combined with the TEM imaging results, the presence of micropores suggests the crystallization of zeolite nuclei into free zeolite crystals. These zeolite crystals penetrate into the larger pores, with some adhering to the mesoporous walls, contributing to the observed increase in monolayer adsorption capacity.

The nitrogen adsorption–desorption isotherm exhibits a typical Type IV profile with a hysteresis loop, which is characteristic of mesoporous materials. This feature indicates that the pore system possesses a three-dimensional (3D) spatial structure, primarily formed due to the disordered arrangement of pores, resulting in an interconnected wormhole-like network. This observation is consistent with the previously analyzed TEM results. Notably, the hysteresis loop is mainly confined within the relative pressure range of P/P_0_ = 0.4–0.6. This localized desorption behavior is typical for disordered, interconnected wormhole-like mesostructures, where the network connectivity affects the capillary evaporation process within a restricted pore size window.

The pore size distribution curve for the MSU-X material (see inset figure in Figure 7) shows that the pores are predominantly around 3.6 nm in diameter. Data from the measurement also reveal that the synthesized MSU-X material has a high specific surface area, with a BET surface area reaching 816 m^2^/g. The large specific surface area is attributed to the 3D wormhole-like structure, as supported by the Transmission Electron Microscopy (TEM) results.

#### 3.3.3. Thermal Stability Evaluation

To examine the thermal stability of the MSU-X material samples, Thermogravimetric Analysis (TGA) combined with Wide-Angle X-ray Diffraction (XRD) at elevated temperatures was used to assess structural transformations and framework integrity. The thermal analysis results of MSU-X are presented in Figure S3 in Supporting Information.

**Figure 8:**
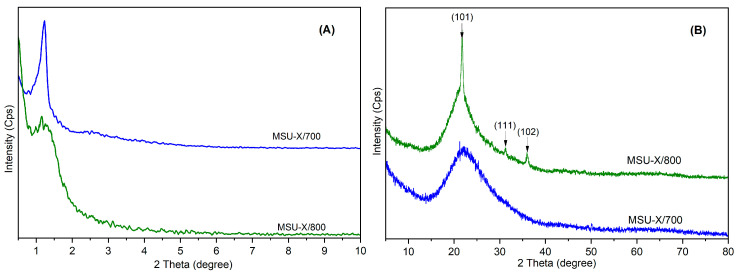
SAXS patterns (**A**) and XRD patterns (**B**) of calcined MSU-X at 700 °C and 800 °C for 3 h.

The TGA profile exhibits a single endothermic event at 89.72 °C, attributed to desorption of physisorbed water, with a corresponding mass loss of 3.08%. To further evaluate the structural stability and potential phase transitions of the MSU-X material, complementary small-angle and wide-angle XRD analyses were conducted (Figure 8A,B).

Wide-angle XRD analysis of samples calcined at 700 °C (MSU-X/700) and 800 °C (MSU-X/800) reveals the framework’s thermal evolution. At 700 °C, the diffraction pattern exhibits a broad hump centered near 22° (2θ), characteristic of the amorphous nature of the walls in mesoporous materials [4,61]. No sharp Bragg reflections emerge, suggesting that the silica walls retain their amorphous phase despite thermal stress.

Upon heating to 800 °C (MSU-X/800), weak diffraction peaks appear at approximately 21.8°, 28.4°, and 36° (2θ), corresponding to incipient cristobalite formation (JCPDS 39-1425) [62,63]. The coexistence of the dominant amorphous halo with minor cristobalite reflections indicates partial phase transformation without wholesale crystallization. The persistence of the broad amorphous background demonstrates that the mesoporous architecture remains largely intact, aligning with established thermochemical pathways where surface-initiated cristobalite nucleation competes with but does not override the kinetic stability of the siloxane network [64].

Comparison of MSU-X/700 and MSU-X/800 profiles reveals a gradual, thermally activated transition following predictable kinetics for amorphous-to-crystalline transformation in silica systems [65].

Overall, the integration of TGA and temperature-resolved XRD demonstrates that MSU-X maintains its amorphous mesostructural framework through 700 °C, with only incipient crystallization at 800 °C. The combination of microscopy and XRD evidence confirms that the material exhibits exceptional thermal resilience—critical for applications as a catalyst support and adsorbent requiring pore accessibility and framework stability under thermal or reactive environments [2,3].

#### 3.3.4. Synthesis Efficiency of MSU-X

The synthesis efficiency of the mesoporous material is critical for establishing the economic viability and robust reproducibility of the method, particularly when utilizing a high-volume waste precursor like Rice Husk Ash (RHA).

Upon identifying the optimized synthetic parameters, three independent batches of MSU-X materials were synthesized under identical conditions to rigorously evaluate the reproducibility of the fabrication protocol. The structural consistency was validated via Small-Angle X-ray Scattering (SAXS) analysis, as shown in Figure 8.

**Figure 9:**
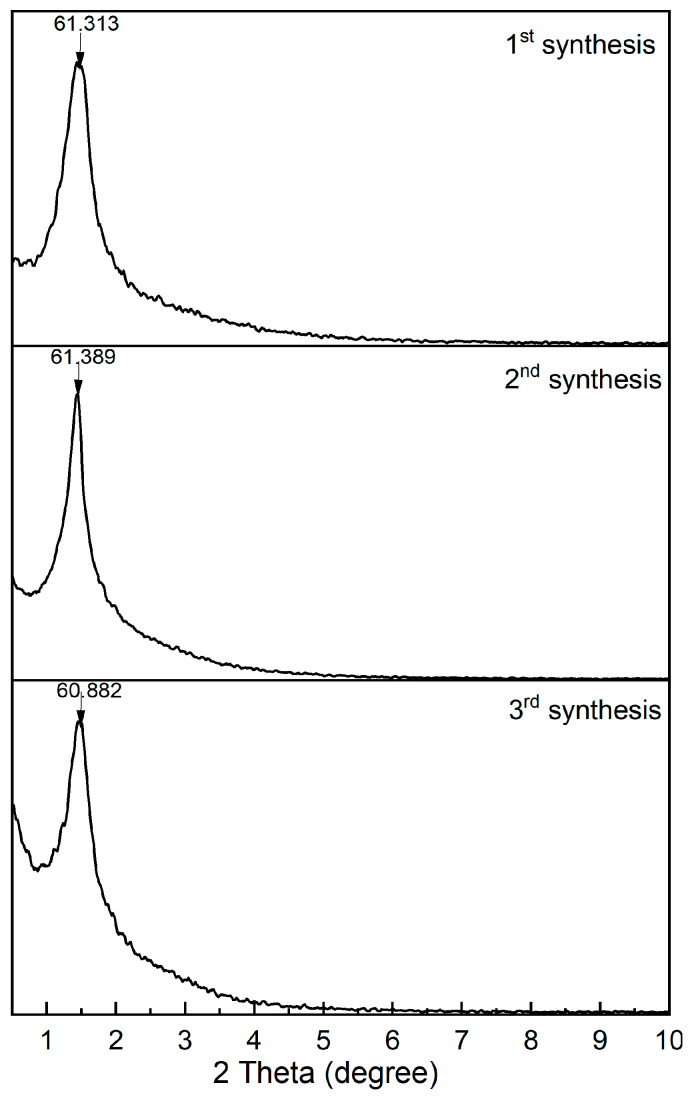
SAXS patterns of MSU-X materials synthesized in three different batches under an aging time of 12 h and a hydrothermal temperature and duration of 60 °C and 96 h, respectively, at pH = 2.

As illustrated in Figure 9, the SAXS profiles of the three distinct batches exhibit sharp, symmetrical, and well-resolved Bragg reflections with highly uniform intensities. This structural coherence allows for a precise assessment of the process repeatability by tracking the interplanar spacing d(100). The d(100) values for all three replicates were calculated from their respective 2θ positions using Bragg’s Law (λ = 2dsinθ), and the consolidated data are summarized in Table 2.

The remarkably low values of Standard Deviation (SD = 0.28 Å) and Relative Standard Deviation (RSD = 0.46%), coupled with the complete absence of secondary or extraneous phases, conclusively demonstrate the exceptional reproducibility and high structural stability of the established sol–gel synthesis pathway.

**Figure 10:**
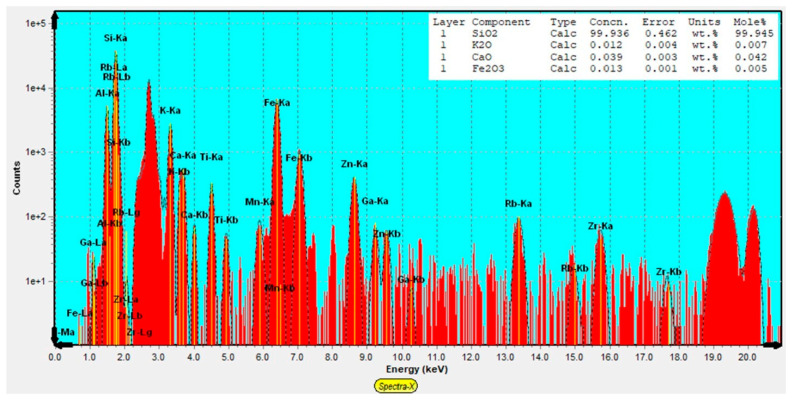
EDXRF spectra of MSU-X materials.

The XRF spectroscopy of synthesized MSU-X is presented in Figure 10.

The theoretical maximum yield was calculated based on the quantitative X-ray Fluorescence (XRF) data in Figure S2 and Figure 10, which established the purity of the RHA precursor as 98.4 wt.% SiO_2_. For the experiment, 5.0 g of RHA were employed, yielding a theoretical maximum available silica mass of:

$$m_{{\mathrm{SiO}}_{2}\;(\mathrm{Theoretical})}\;=\;5.0\;g\;\times \;0.984\;=\;4.92\;g$$

Following the two-step alkali dissolution and acid-catalyzed condensation, the recovered mass of the calcined MSU-X product was 4.71 g, and the percentage of SiO_2_ was approximately 99.936%. The synthesis yield (Y) was thus determined as the ratio of the recovered product mass to the theoretical maximum SiO_2_ mass in the precursor:

$$Y\left(\%\right)\;=\left(\frac{m_{\mathrm{MSU-X}}}{m_{{\mathrm{SiO}}_{2}\;(\mathrm{Theoretical})}}\right)\;\times \;100\%$$

$$Y\left(\%\right)=\left(\frac{4.71\;\times \;0.994\;g}{4.92\;g}\right)\;\times \;100\%=95.5\%$$

This remarkably high yield of 95.5% places the reaction efficiency on par with, or exceeding, those reported for the synthesis of ordered mesoporous materials (like MSU-X and MCM-41) using expensive, high-purity commercial precursors such as tetraethyl orthosilicate [1,5,8,15]. Furthermore, the overall efficiency of 94.2% (based on the total mass of the initial 5.0 g RHA used) is significantly superior to the typical 80–90% conversion rates often reported in the literature for the initial alkaline extraction of silica from low-cost RHA (Table 3).

The success of achieving a 95.5% yield validates that (i) the initial alkaline leaching process was near-complete, effectively transferring the majority of the 4.92 g SiO_2_ into the soluble sodium silicate form, and (ii) the subsequent condensation and assembly steps proceeded with minimal loss, resulting in a highly efficient mass conversion from the extracted silicate species to the final structured MSU-X framework. This efficiency underscores the optimized nature of the synthesis protocol, making it a sustainable and cost-effective route for large-scale production of high-purity mesoporous silica.

### 3.4. Z8/MSU-X Characterization

To introduce ZIF-8 framework precursors in the form of nucleation seeds onto the surface of MSU-X mesoporous silica—with the aim of generating a high density of hydrophobically active adsorption sites for the target organochlorine pollutant DDT—the resulting Z8/MSU-X composite was subjected to comprehensive structural and compositional characterization by powder X-ray diffraction (XRD), Fourier-transform infrared spectroscopy (FTIR), and scanning electron microscopy (SEM). The three analytical techniques provide complementary and mutually reinforcing evidence, which is discussed in an integrated manner below.

**Figure 11:**
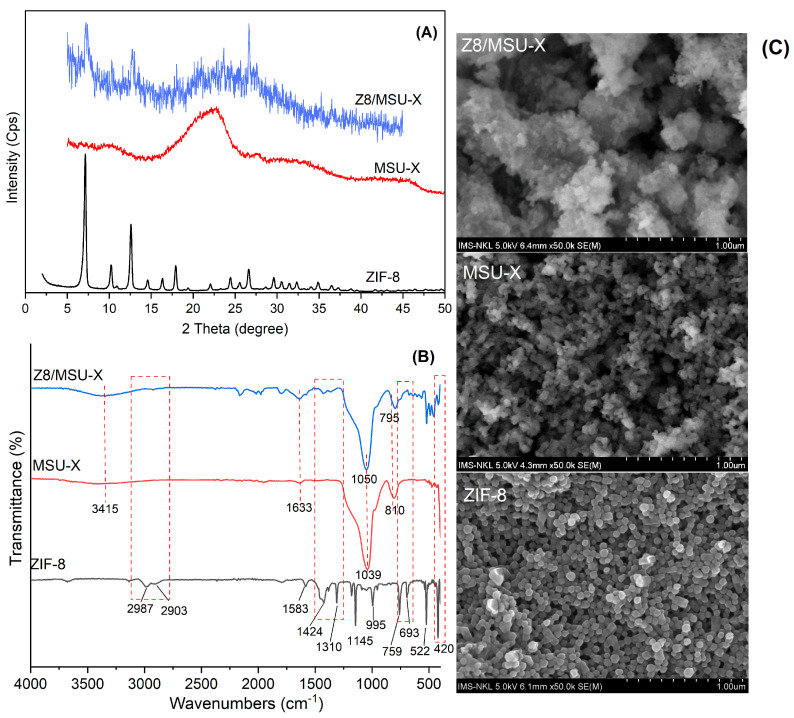
XRD pattern (**A**), FTIR spectra (**B**) and SEM images of synthesized MSU-X, ZIF-8, and Z8/MSU-X (**C**).

The wide-angle XRD pattern of the solvothermally synthesized bulk ZIF-8 reference (Figure 11A) displays six well-resolved diffraction peaks at 2θ = 7.1°, 10.2°, 12.6°, 16.2°, 24.5°, together with their higher-angle counterparts, indexed to the (011), (002), (112), (022), (013), and (233) reflection planes of the sodalite-type cubic framework, in full agreement with the literature [33]. Pristine MSU-X, as expected for an amorphous wormhole-channel silica, produces only a broad, featureless background-scattering envelope without any sharp Bragg reflections, confirming the absence of long-range crystalline periodicity. For Z8/MSU-X, no diffraction peaks attributable to crystalline ZIF-8 are discernible above the noise level of the measurement. This result should not, however, be interpreted in isolation: the detection limit of laboratory XRD precludes identification of crystalline domains below approximately 3–5 nm, and low ZIF-8 loadings dispersed across the silica surface would similarly fall below the threshold of detection. The XRD data therefore establish an upper bound on the crystallite size of any ZIF-8 phase present but cannot alone distinguish between complete absence of ZIF-8 and the presence of sub-nanometric, amorphous coordination clusters. Definitive structural interpretation thus requires the complementary molecular-level evidence provided by FTIR.

The FTIR spectra of ZIF-8, MSU-X, and Z8/MSU-X are presented in Figure 11B. The spectrum of bulk ZIF-8 exhibits N–H stretching vibrations of the protonated 2-methylimidazole ligand in the region 2903–2987 cm^−1^, alongside in-plane imidazolate ring vibrations at 1308–1423 cm^−1^, out-of-plane bending modes at 995–1145 cm^−1^, in-plane C–N stretching at 693–759 cm^−1^, and, most diagnostically, a Zn–N stretching band at 420 cm^−1^, all consistent with the established vibrational signature of the ZIF-8 coordination framework [34,35]. The spectrum of pristine MSU-X is dominated by a broad O–H stretching envelope centered at 3415 cm^−1^ from surface silanol groups and adsorbed water, a Si–OH bending mode at 1633 cm^−1^, and the characteristic asymmetric Si–O–Si and Si–O stretching bands at 1039 and 810 cm^−1^, respectively [66].

The FTIR spectrum of Z8/MSU-X reveals several features that, taken collectively, provide molecular-level evidence for successful ZIF-8 framework grafting. First, the N–H stretching bands present in free 2-methylimidazole and bulk ZIF-8 are no longer detectable, consistent with complete deprotonation of the imidazole ligand and formation of Zn^2+^–imidazolate coordination bonds [67]. It is acknowledged that signal masking by the broad MSU-X Si–OH envelope in the same spectral region cannot be fully excluded on the basis of FTIR alone; complementary XPS analysis of the N 1s binding energy region would provide unambiguous confirmation of the coordination state of nitrogen and is recommended for future work. Second, out-of-plane imidazolate ring bending modes are retained in the composite spectrum, and spectral deconvolution of the 1000–1100 cm^−1^ region reveals a shoulder superimposed on the dominant Si–O–Si stretching band, attributable to C–N stretching within the imidazolate ring—a contribution absent in the spectrum of pristine MSU-X. Third, and most critically, a Zn–N stretching band at 417 cm^−1^ is clearly resolved in the spectrum of Z8/MSU-X, providing direct evidence for Zn–N coordination at the composite surface. The slight red-shift relative to bulk ZIF-8 (420 cm^−1^) is consistent with a modified local coordination environment arising from interfacial anchoring of the framework to the silica surface, rather than the formation of a bulk periodic crystal lattice.

Interpreted in conjunction with the XRD data, the FTIR evidence supports the conclusion that the ZIF-8 precursor in Z8/MSU-X exists as a chemically intact but structurally disordered coordination framework—retaining the essential Zn–N bonding motif of ZIF-8—anchored to the MSU-X surface in a sub-crystalline, seed-stage configuration rather than as discrete, long-range-ordered crystalline domains.

The SEM image of the bulk ZIF-8 reference (Figure 11C) shows well-defined rhombic dodecahedral crystallites with a narrow particle size distribution centered at 98 ± 12 nm (measured from n = 50 particles), consistent with the expected morphology of solvothermally synthesized ZIF-8. Pristine MSU-X appears as agglomerated near-spherical particles of comparable size (average diameter: 95 ± 15 nm). The SEM image of Z8/MSU-X reveals a marked change in surface texture: particle boundaries become less distinct and the surface adopts a roughened, conformal coating morphology relative to bare MSU-X, qualitatively consistent with deposition of a ZIF-8 framework layer.

The convergent evidence from XRD, FTIR, and SEM-EDX collectively establishes that the synthetic protocol employed successfully anchors a chemically active ZIF-8 coordination framework—preserving the Zn–N bonding environment and imidazolate ligand integrity essential for hydrophobic guest interactions—onto the mesoporous MSU-X surface in a uniformly dispersed, sub-crystalline state. The deliberate absence of bulk ZIF-8 crystallization is a structurally advantageous outcome: it ensures that the ZIF-8 framework is maximally accessible at the composite surface rather than confined within the interior of opaque crystalline domains, thereby maximizing the density and accessibility of active adsorption sites for DDT uptake, as subsequently demonstrated by the adsorption performance data discussed in the next section.

**Figure 12:**
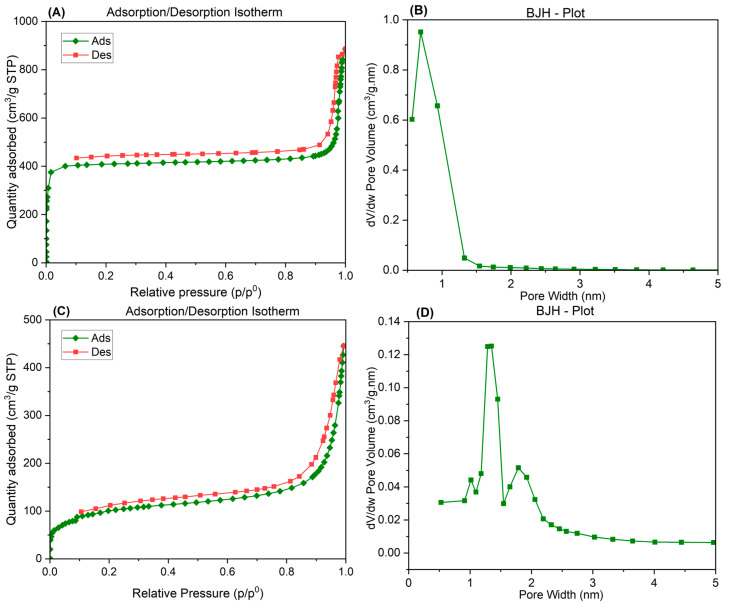
N_2_ adsorption–desorption BET diagrams and BJH plots of ZIF-8 (**A**,**B**) and Z8/MSU-X (**C**,**D**).

Figure 12 presents nitrogen adsorption–desorption isotherms and pore size distribution curves of ZIF-8 (Figure 12A,B) and Z8/MSU-X (Figure 12C,D).

The isotherms exhibit characteristic Type IV profiles with Type H4 hysteresis loops, indicative of microporous architectures featuring slit-shaped pore geometries. For the pristine ZIF-8 sample, the pronounced nitrogen uptake at low relative pressures (P/P_0_ < 0.1) is diagnostic of micropore filling, while the adsorption–desorption hysteresis observed in the relative pressure range of 0.4–0.9 is attributable to capillary condensation within narrow interparticle slit pores. The material exhibits a pore diameter of 0.8 nm and a BET-specific surface area of 1254 m^2^/g.

Figure 12C,D reveals that the integration of ZIF-8 into the MSU-X support yields a composite (Z8/MSU-X) that retains a pore system situated at the micropore–mesopore boundary, as evidenced by a contraction of the pore diameter to approximately 1.8–2.0 nm relative to the parent MSU-X. This structural narrowing is accompanied by a reduction in total pore volume from 0.8 to 0.6 cm^3^/g and a decrease in BET surface area to 342 m^2^/g, collectively attributable to partial occlusion of the mesoporous channels by ZIF-8 crystallites nucleated within or at the entrance of the MSU-X pore network.

### 3.5. MSU-X as Support

**Figure 13:**
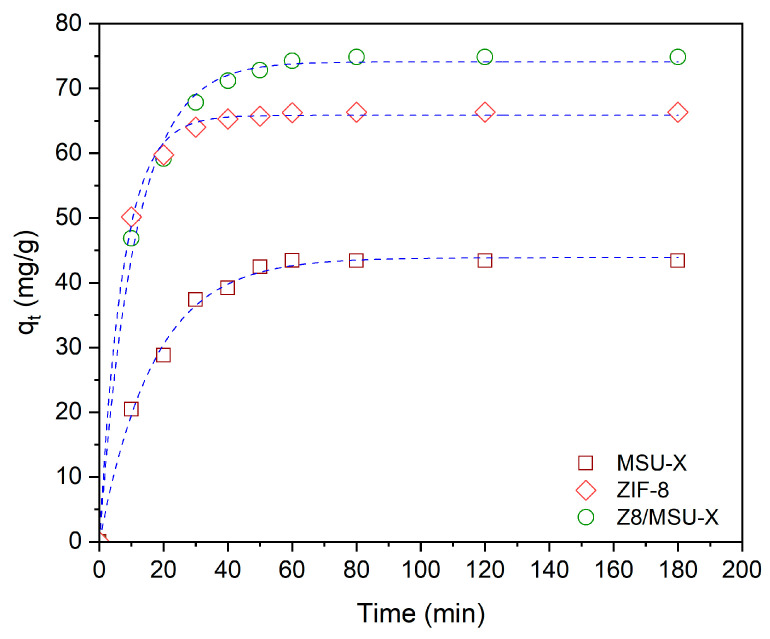
Effect of contact time on adsorption capacity. Dashed lines indicate curves fitted using nonlinear PFO kinetic model for DDT on MSU-X, ZIF-8, and Z8/MSU-X.

The effect of contact time on adsorption performance was evaluated to provide insight into the functional role of MSU-X as a support material (Figure 13).

The influence of contact time on the DDT adsorption capacity onto MSU-X, ZIF-8, and Z8/MSU-X materials is illustrated in Figure 13. To evaluate the experimental data, the pseudo-first-order (PFO) kinetic model for DDT adsorption onto MSU-X, ZIF-8, and Z8/MSU-X materials was applied, with the corresponding parameters summarized in Table 4. The coefficient of determination (R^2^) and root mean square error (RMSE) values demonstrate that the adsorption kinetics for all three materials are well described by the PFO model, supporting a process governed primarily by physical interactions. This selection is strongly validated by the recent work of K.H. Chu et al. [68], which demonstrated that a more appropriate model representing intraparticle diffusion-limited transport is mathematically analogous to the PFO framework (specifically, Equation (22) in their study), thereby justifying its presentation as the sole kinetic model. Conversely, the pseudo-second-order (PSO) model was excluded from the discussion due to its poor fit and lack of physical significance for the current system.

The experimental results show that the adsorption of DDT onto MSU-X, ZIF-8, and Z8/MSU-X reached 43.85 mg/g, 65.83 mg/g, and 74.10 mg/g, respectively, within 60 min. Pristine ZIF-8 exhibited a higher apparent rate coefficient (k_1_ = 0.136 h^−1^) compared to the Z8/MSU-X composite (k_1_ = 0.089 h^−1^). This indicates that the adsorption on unsupported ZIF-8 proceeds more rapidly, which is attributed to the immediate accessibility of the active imidazole sites on its external surface. For the Z8/MSU-X composite, the integration of ZIF-8 into the MSU-X framework alters the mass transport path, resulting in a lower initial rate coefficient.

Despite the lower kinetic rate, the critical role of the MSU-X support is reflected in the significantly enhanced total uptake of the composite. In pristine ZIF-8, the rapid equilibrium is limited by particle agglomeration, which buries a fraction of the active imidazole nodes within the bulk crystal aggregates. The mesoporous MSU-X framework functions effectively as an anti-agglomeration scaffold. By providing a high-surface-area matrix, it facilitates the highly dispersed growth of smaller ZIF-8 crystals, thereby increasing the net exposure and utilization efficiency of the active adsorption sites.

Consequently, the Z8/MSU-X composite achieved an adsorption capacity of 74.10 mg/g, effectively removing nearly all the DDT initially present in the solution. Under these specific experimental conditions, the system operates within a reagent-limited regime rather than a capacity-saturated boundary. The efficient depletion of the available adsorbate within the explored concentration range conclusively validates that the MSU-X support prevents active site loss, thereby maximizing the structural performance of the integrated ZIF-8 phases.

## 4. Conclusions

This work demonstrates that agricultural silica waste—specifically rice husk ash purified to ~98.4 wt% SiO_2_ by acid leaching and controlled calcination—constitutes a fully competent precursor for the direct sol–gel assembly of wormhole-like MSU-X mesoporous frameworks under unmodified standard synthesis conditions. The central finding, carrying both mechanistic and practical significance, is that the compositional heterogeneity intrinsic to biogenic feedstocks does not necessitate adjusted reaction environments: retention of canonical parameters (pH 2, Si/Tergitol = 8, 60 °C, 96 h) was sufficient to drive near-quantitative silicate-to-mesostructure conversion, yielding a synthesis efficiency of ~95.5%—a value on par with, or exceeding, those reported for reagent-grade TEOS and commercial sodium silicate. The optimized RHA-derived MSU-X exhibits a BET surface area of 816 m^2^ g^−1^ and a uniform wormhole-type pore network centered at ~3.6 nm. The existence of the disordered wormhole-like mesostructure of the as-synthesized material is maintained up to 700 °C, with only incipient cristobalite nucleation emerging near 800 °C—benchmarks that rival state-of-the-art mesoporous silicas from conventional precursors. EDXRF tracking across the full synthesis sequence revealed progressive silica enrichment from 98.4 wt% in RHA to 99.9 wt% in the calcined product, providing direct evidence of impurity expulsion during sol–gel condensation. When deployed as a mesoporous scaffold for ZIF-8 nucleation, the role of the MSU-X support extends beyond providing structural reinforcement for the composite framework; it also potentially alters the adsorption characteristics through its three-dimensionally interconnected wormhole-like mesoporous network. Under the specific experimental conditions investigated, the Z8/MSU-X composite demonstrated an enhanced DDT adsorption capacity (~74.10 mg g^−1^) compared to pristine ZIF-8 (~65.83 mg g^−1^). This enhancement may be attributed to the improved accessibility of active sites within the mesoporous architecture or a synergistic effect arising from capillary forces coupled with interactions at the ZIF-8 nodes. Collectively, these findings establish a quantitative, parameter-resolved synthesis framework that closes a critical knowledge gap: well-controlled sol–gel processing can faithfully translate to non-purified biogenic silica without compromising mesostructural fidelity, thermal resilience, or functional performance—providing a scientifically rigorous and economically viable pathway toward circular-economy-aligned production of high-performance mesoporous silica for catalytic, environmental, and energy-related applications.

## Figures and Tables

**Table 1:** Synthesis conditions of MSU-X.

Sample	Synthesis Conditions				
	Aging Time (h)	Hydrothermal Temperature (°C)	Hydrothermal Time (h)	pH	Si/Ter Molar Ratio
MSU-X/A8	8	60	96	2	8
MSU-X/A12	12	60	96	2	8
MSU-X/A24	24	60	96	2	8
MSU-X/T30	12	30	96	2	8
MSU-X/T45	12	45	96	2	8
MSU-X/T60	12	60	96	2	8
MSU-X/H48	12	60	48	2	8
MSU-X/H72	12	60	72	2	8
MSU-X/H96	12	60	96	2	8
MSU-X/pH2	12	60	96	2	8
MSU-X/pH7	12	60	96	7	8
MSU-X/pH9	12	60	96	9	8
MSU-X/S6	12	60	96	2	6
MSU-X/S8	12	60	96	2	8
MSU-X/S12	12	60	96	2	12

**Table 2:** Structural parameters and statistical reproducibility of the triplicate MSU-X syntheses.

Sample	2 Theta (°)	d(100) (Å)	SD (Å)	RSD (%)
1st synthesis	1.440	61.131		
2nd synthesis	1.438	61.389	0.28	0.46
3rd synthesis	1.450	60.882		

(Note: SD and RSD values represent the collective statistical metrics calculated across all three independent runs).

**Table 3:** Efficiency of silica mesoporous synthesis in published studies.

References	Year	Method	Yield	Conditions
**TEOS-based**				
Beck et al. [1]	1992	MCM-41 from TEOS	85–92%	Classic protocol
Kim et al. [8]	1998	MSU-X from TEOS	88–94%	Non-ionic surfactant
Zhao et al. [5]	1998	SBA-15 from TEOS	90–95%	P123 template
Gebretatios et al. [15]	2023	Review (TEOS-based)	85–95%	Various methods
**RHA Extraction (most recent)**				
Nugraha et al. [27]	2025	Review: NaOH extraction	82–92%	1–5 M NaOH, 60–100 °C
Juárez et al. [17]	2025	Reflux method	84–89%	NaOH reflux
Gebretatios et al. [22]	2024	Optimized alkaline	86–91%	Optimized conditions
Dhiman et al. [31]	2025	Review: Agri-waste	78–88%	Various protocols
Oliveira et al. [28]	2023	Review: Standard conditions	80–90%	Literature survey
Zhao et al. [25]	2024	Sol–gel method	83–90%	2 M NaOH, 80 °C, 2 h
Nzereogu et al. [21]	2023	Systematic review	75–92%	Most: 85–90%
Florek et al. [32]	2025	Circular economy review	80–88%	Sustainable methods

**Table 4:** Pseudo-first- and pseudo-second-order rate constants for adsorption of DDT onto MSU-X, ZIF-8, and Z8/MSU-X.

Adsorbent	Pseudo-First-Order			
	q_e_ (mg/g)	k_1_ (1/h)	R^2^	RMSE
MSU-X	43.85	0.059	0.996	0.316
ZIF-8	65.83	0.136	0.998	0.296
Z8/MSU-X	74.10	0.089	0.996	0.981

## Data Availability

The original contributions presented in this study are included in the article/Supplementary Materials. Further inquiries can be directed to the corresponding author.

## Supplementary Materials

The following supporting information can be downloaded at: https://www.mdpi.com/article/10.3390/nano16120748/s1, Figure S1. FTIR spectra (A), XRD pattern (B) of rice husk (RH) and rice husk ash (RHA). Figure S2. EDXRF spectra of rice husk ash. Figure S3. TGA diagram (A) of the MSU-X material.
