# Supplementary material for: Biogenic Silica as a Direct Sol–Gel Precursor for High-Efficiency MSU-X Mesostructure Assembly: Closing the Loop from Rice Husk Waste to Functional Wormhole Frameworks
Source: Nanomaterials (Basel). 2026 Jun 15;16(12):748. doi: 10.3390/nano16120748 (PMC13305862; doi:10.3390/nano16120748)
Supplement: Supplementary file 1 [file nanomaterials-16-00748-s001.zip › nanomaterials-4316530-supplementary.pdf]

Supporting information

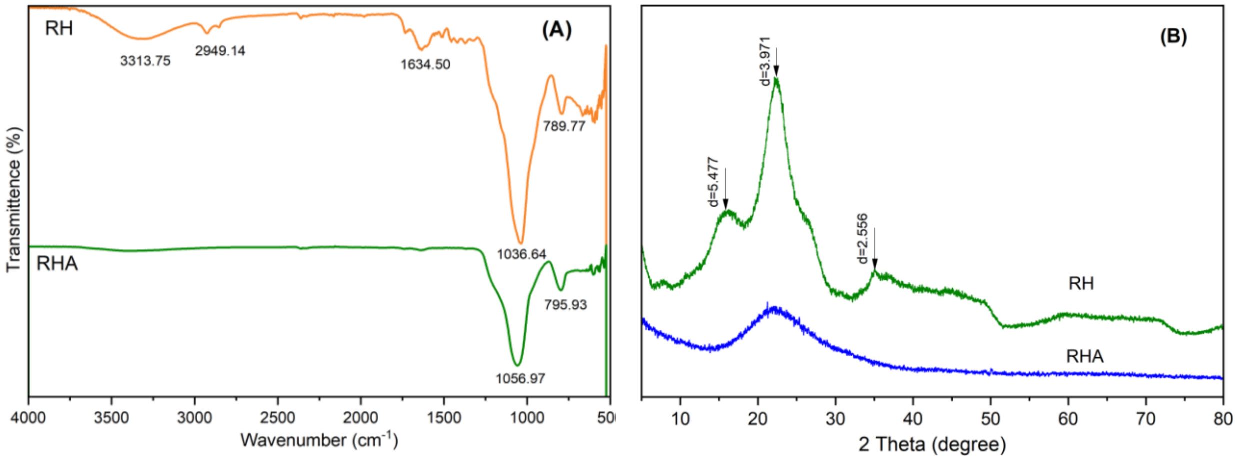

Figure S1. FTIR spectra (A), XRD pattern (B) of rice husk (RH) and rice husk ash (RHA).

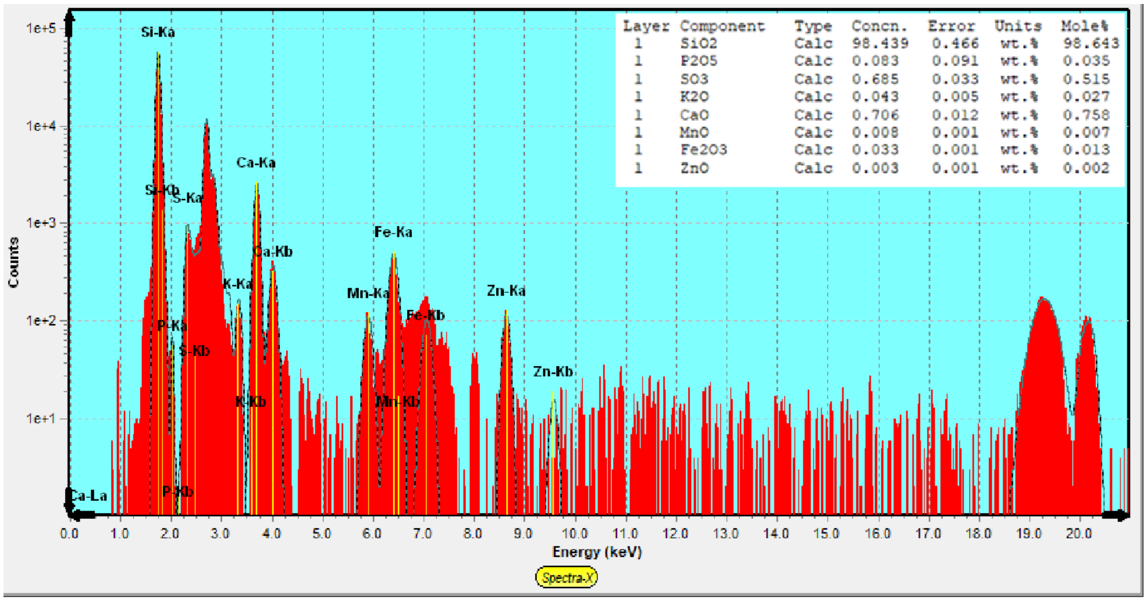

Figure S2. EDXRF spectra of rice husk ash.

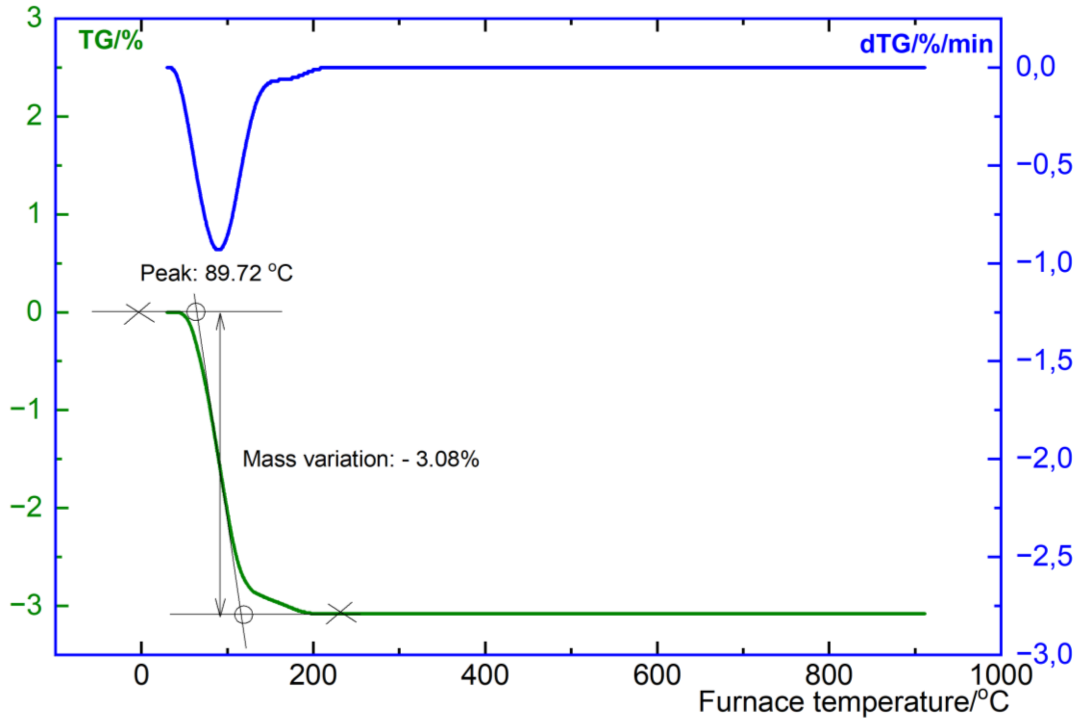

Figure S3. TGA diagram (A) of the MSU-X material
